# Supplementary material for: High Value Utilization of Waste Wood toward Porous and Lightweight Carbon Monolith with EMI Shielding, Heat Insulation and Mechanical Properties
Source: Molecules. 2023 Mar 8;28(6):2482. doi: 10.3390/molecules28062482 (PMC10056734; doi:10.3390/molecules28062482)
Supplement: Supplementary file 1 [file molecules-28-02482-s001.zip › molecules-2212663-supplementary.pdf]

Supporting Information

EMI shielding measurement: The schematic diagram of the EMI shielding performance of samples in X-band frequency (8.2-12.4 GHz) is shown in **Figure S2**. The set-up consists of a scalar network analyzer, sweep oscillator, three detectors, four coaxial to waveguide adapters and two waveguide directional couplers connected reflectometer. In the set-up, detector R is used to measure the incident wave; while detectors A and B are used to measure the reflected and transmitted waves. The sample to be tested was placed between the directional coupler and the adapter. The samples prepared for EMI SE characterization are larger than the window of the directional coupler. The directional coupler opening is 22.86 mm·× 10.16 mm.

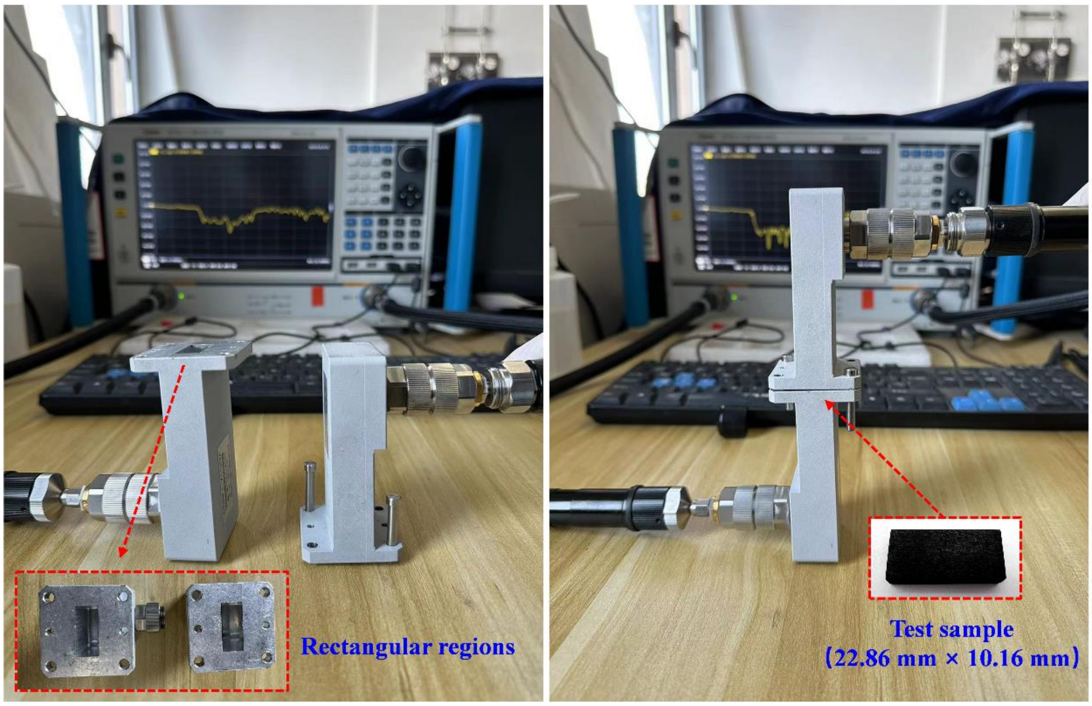

Figure S1. Pictures of the Vector Network Analyzer (Agilent Technologies N5063A, USA)

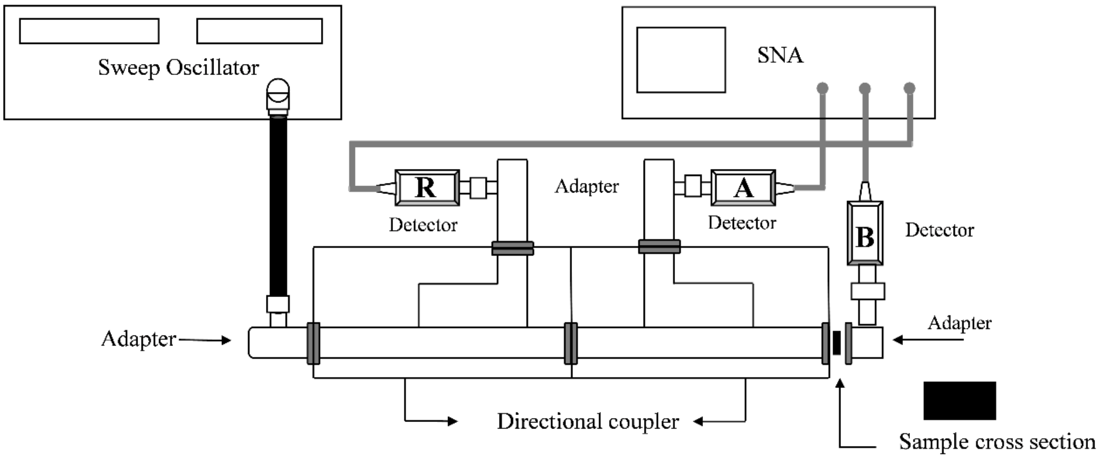

Figure S2. Schematic sketch shows the instrument used to evaluate the EMI SE.
